# Determinants of overweight or obesity among ever-married adult women in Bangladesh

**DOI:** 10.1186/s40608-016-0093-5

**Published:** 2016-03-01

**Authors:** Haribondhu Sarma, Nazmus Saquib, Md Mehedi Hasan, Juliann Saquib, Ahmed Shafiqur Rahman, Jahidur Rahman Khan, Md Jasim Uddin, Mark R. Cullen, Tahmeed Ahmed

**Affiliations:** Nutrition and Clinical Services Division, icddr,b, Mohakhali, 1212, GPO Box 128, Dhaka 1000 Dhaka, Bangladesh; Sulaiman AlRajhi College, Qassim, Kingdom of Saudi Arabia; Research, Learning and Evaluation, Helen Keller International, Dhaka, Bangladesh; Department of Family and Community Medicine, Qassim University, Qassim, Kingdom of Saudi Arabia; Health Systems and Population Studies Division, icddr,b, Mohakhali, 1212 GPO Box 128, Dhaka 1000 Dhaka, Bangladesh; Division of General Medical Disciplines, Stanford University School of Medicine, Stanford, USA

**Keywords:** Overweight, Obesity, Nutritional Status, Women, Bangladesh

## Abstract

**Background:**

The prevalence of overweight and obesity is increasing in Bangladesh. It is higher among Bangladeshi women than among men. This study was conducted to assess a host of demographic and socioeconomic correlates of overweight and obesity, separately for the urban and rural women of Bangladesh.

**Methods:**

We used data from the Bangladesh Demographic and Health Survey (BDHS) 2011. The BDHS provides cross-sectional data on a wide range of indicators relating to population, health, and nutrition. We analyzed nutrition-related data to identify the factors associated with being overweight or obese among ever-married women aged 18–49 years.

**Results:**

Of 16,493 women, about 18 % (95 % CI 17 · 80–18 · 99) were overweight or obese. Unemployed urban women were at 1 · 44 (95 % CI 1 · 18–1 · 76, *p* < 0 · 001) times higher risk of being overweight or obese than those women who were involved in manual-labored work. Watching television at least once a week was another significant predictor among urban women (OR 1 · 49; 95 % CI 1 · 24–1 · 80; *p* < 0 · 001) and rural women (OR 1 · 31; 95 % CI 1 · 14–1 · 51; *p* < 0 · 001). Household wealth index and food security were also strongly associated with overweight or obesity of both rural and urban women.

**Conclusions:**

The findings of the study indicate that a large number of women in Bangladesh are suffering from being overweight or obese, and multiple factors are responsible for this including, older age, being from wealthy households, higher education, being from food-secured households, watching TV at least once a week, and being an unemployed urban woman. Given the anticipated long-term effects, the factors that are associated with being overweight or obese should be considered while formulating an effective intervention for the women of Bangladesh.

**Electronic supplementary material:**

The online version of this article (doi:10.1186/s40608-016-0093-5) contains supplementary material, which is available to authorized users.

## Background

Historically, overweight and obesity were largely problems in high-income countries; however, their prevalence is increasing day by day in many low- and middle-income countries across the world [1]. Overweight and obesity are directly correlated with the outcomes of many non-communicable diseases (NCDs), such as type II diabetes (diabetes mellitus), ischemic heart disease, stroke, hypertensive heart disease, and many others [1–3]. At present, NCDs are the predominant cause of deaths in Bangladesh. The proportion of deaths due to NCDs increased from 8 % in 1986 to 68 % in 2006 [4]. Results of a recent review of studies on NCDs in Bangladesh showed that rates of both type II diabetes and cardiovascular diseases have steadily increased between 1995 and 2010 [5]. On the other hand, the consequences of being overweight or obese may disproportionately affect women compared to men [6]. Women with abdominal obesity are susceptible to type II diabetes, and diabetic women are disproportionately at a higher relative risk of coronary heart disease than diabetic men [6]. Moreover, obesity substantially increases the risk of several major cancers among women, such as postmenopausal breast cancer and endometrial cancer [7].

Evidence shows that the prevalence of overweight and obesity is higher among women than among men in Bangladesh [8], and the sex difference in the prevalence of obesity is higher for centripetal than general obesity [5]. Little information is available on correlates of obesity in women, particularly reproductive, lifestyle, and socioeconomic indicators. There is some indication that physical inactivity in women may play an important role in increasing overweight or obesity. According to the World Health Organization, the prevalence of physical inactivity is higher among Bangladeshi women than among women of other Asian countries [9]. The prevalence of overweight and obesity also differs highly between the rural and urban women of Bangladesh. The BDHS, described below, collects data on body-weight and associated covariates, using a nationally-representative sample. This has allowed us to use the most recent 2011 survey to examine the correlates of overweight and obesity in urban and rural women separately [10].

## Methods

Data for the present study were drawn from the BDHS 2011. This nationally- representative household survey used a multistage-stratified cluster-sampling design and provides cross-sectional data on various indicators relating to population, health, and nutrition. The survey, typically carried out every five years, using a well-described and standardized methodology, usually covers ever-married women, men, and children under five years old. Details about the sampling, survey design, survey instruments, and quality control are described in the report of the BDHS 2011 [10]. Trained personnel collected anthropometric data (height and weight) using standardized procedures: weight was measured using a solar-powered scale (UNICEF electronic scale or Uniscale) with accuracy to 0 · 1 kg, and height was measured using a standardized measuring board with accuracy to 0 · 1 cm.

**Fig. 1:**
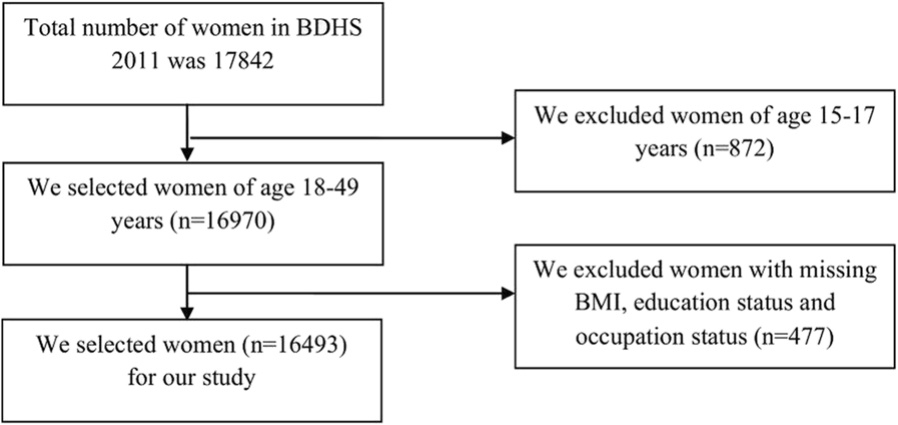
Flow-chart, extracted data from BDHS 2011

Informed consent was obtained from participants while interviewing them. The Institutional Review Board of the Bangladesh Medical Research Council approved the BDHS 2011 survey. This open-access dataset is available to researchers. In the dataset, participants have been identified with unique numbers but not with any personal information. The process of extracting data was as follows: we first extracted data from the women file of the BDHS 2011, and then we extracted our required variables. We then selected women aged 18–49 years and those who had a body mass index (BMI) value. We checked the missing values of all variables considered in this paper. We excluded women for whom there was missing information on height and/or weight and women for whom a BMI could not be estimated because they were pregnant or had given birth in the preceding two months. We also excluded women from whom there was missing information on education and occupation status (Fig. 1). In addition, we imputed “Age at first birth” variable using the mean imputation procedure.

Our analysis was limited to women aged 18–49 years, as in Bangladesh, ever-married adult women are considered from the age of 18 years and BDHS 2011 did not collect information of women aged above 49 years. BMI was defined as weight in kg divided by height in meter squared (kg/m^2^). According to the WHO definition, a BMI of <25 kg/m^2^ was not considered to be overweight or obesity while a BMI of ≥25 kg/m^2^ was considered to be overweight and obesity. In our study, the prevalence of overweight and obesity was considered the primary outcome of interest (dependent variable). We used 14 predictor variables, such as type of residence (rural and urban), administrative division (Dhaka, Chittagong, Rajshahi, Khulna, Barisal, Rangpur, and Sylhet), number of household members (categorized as 1–2, 3–4, and ≥5), wealth index (poorest, poorer, middle, richer, and richest), status of food security of households [categorized as food secure (according to BDHS 2011, ever-married women who reported that they did not experience any food insecurity, i.e., lack of access, or had to worry about food were considered as belonging to food- secure households [10]) and food insecure], status of contraceptive use (never user, current user, and past user), menopausal status (not menopause and menopause), number of living children (categorized as 0 ^ref.^, 1–2, 3–4, and ≥5), current marital status (married/living with husband, and widowed/divorce/separated), educational status (no education, primary completed, secondary completed, higher secondary completed or more), occupation (jobs that required manual labor, jobs that required mostly sitting, and unemployed/housewife), age in years (categorized as 18–22, 23–27, 28–32, 33–37, 38–42, and ≥43), and watching TV at least once a week (yes and no). The SPSS software (version 20), STATA (version 16), and R (version 3 · 0 · 0) were used for analyzing data.

Analysis of data began with descriptive analysis to know the frequencies and percentages of the variables of interest. We then performed unadjusted (i.e. simple) binary logistic regressions to assess the association of each independent variable with the dependent variable (i.e. overweight and obesity). Finally, we built adjusted (i.e. multiple binary logistic regression) models of overweight and obesity by including all the independent variables which were found to be significant in the unadjusted model. We have used stepwise regression technique for selecting a subset of variables that are useful in predicting a response (overweight and obesity). We also checked multicollinearity (correlation between independent variables) by using generalized variance inflation factor (GVIF), and confirmed that there was no multicollinearity (GVIF <4.0) in adjusted models. Adjusted models for urban and rural women were developed separately because research indicated a difference in urban–rural overweight or obesity. As a sensitivity procedure, we repeated our analyses after we used different cut-offs (BMI ≥ 23 kg/m^2^ and ≥ 27 kg/m^2^) for definitions of overweight and obesity.

## Results

Of the 16,493 women identified in this analysis, 57 % were less than 32 years old; and 94 % were married and living with their husbands. Urban women constituted 35 %. The samples are well-represented among all the seven geographical divisions of Bangladesh. The majority (60 %) of the respondents was living in households of five or more members, 65 % fell under the category of food-secure households, 27 % (7 % urban and 20 % rural) had no formal education, and 85 % (28 % urban and 57 % rural) did not work outside the house as they were either unemployed or housewives. Eighty eight percent had their first child when they were between the ages of 13 and 22 years. About 50 % watched TV at least once a week. The overall prevalence of overweight and obesity was 18 %, where overweight was 15 % and obese was 3 % (Table 1).

**Table 1:** Frequency and percentage distribution of overweight or obesity of Bangladeshi women and other selected variables

	Urban			Rural		
Variables with categories	n	%	95 % CI	n	%	95%CI
Overweight or obesity status of women						
BMI < 25 (Not overweight or obese)	4083	24 · 75	24 · 09–25 · 41	9376	56 · 85	56 · 08–57 · 60
BMI ≥ 25 & BMI < 30 (Overweight)	360	2 · 18	1 · 96–2 · 41	188	1 · 14	0 · 98–1 · 31
BMI ≥ 30 (Obese)	1318	7 · 99	7 · 57–8 · 41	1168	7 · 08	6 · 68–7 · 48
Age (years) of women						
18–22	1020	6 · 18	5 · 81–6 · 56	2069	12 · 55	12 · 03–13 · 06
23–27	1194	7 · 24	6 · 84–7 · 64	2251	13 · 65	13 · 11–14 · 18
28–32	1042	6 · 32	5 · 94–6 · 70	1861	11 · 28	10 · 79–11 · 78
33–37	804	4 · 88	4 · 55–5 · 21	1530	9 · 28	8 · 83–9 · 73
38–42	814	4 · 94	4 · 60–5 · 27	1451	8 · 80	8 · 36–9 · 24
43+	887	5 · 38	5 · 03–5 · 73	1570	9 · 52	9 · 06–9 · 98
Current marital status						
Married and living with husband	5365	32 · 53	31 · 80–33 · 24	10060	60 · 99	60 · 24–61 · 74
Widowed/divorced/separated	396	2 · 40	2 · 16–2 · 64	672	4 · 07	3 · 77–4 · 38
Division						
Barisal	611	3 · 71	3 · 41–3 · 99	1252	7 · 59	7 · 18–8 · 00
Chittagong	982	5 · 95	5 · 59–6 · 32	1694	10 · 27	9 · 80–10.74
Dhaka	1235	7 · 49	7 · 08–7 · 90	1592	9 · 65	9 · 19–10 · 11
Khulna	857	5 · 20	4 · 85–5 · 42	1613	9 · 78	9 · 32–10 · 24
Rajshahi	800	4 · 85	4 · 52–5 · 19	1584	9 · 60	9 · 15–10 · 06
Rangpur	656	3 · 98	3 · 67–4 · 28	1645	9 · 97	9 · 51–10 · 44
Sylhet	620	3 · 76	3 · 46–4 · 06	1352	8 · 20	7 · 77–8 · 63
No · of household members						
1–2	276	1 · 67	1 · 47–1 · 87	450	2 · 73	2 · 48–2 · 98
3–4	2143	12 · 99	12 · 47–13 · 52	3648	22 · 12	21 · 49–22 · 75
5+	3342	20 · 26	19 · 64–20 · 89	6634	40 · 22	39 · 46–40 · 97
Wealth index of households						
Poorest	411	2 · 49	2 · 25–2 · 74	2456	14 · 89	14 · 34–15 · 43
Poorer	405	2 · 46	2 · 22–2 · 70	2629	15 · 94	15 · 37–16 · 51
Middle	600	3 · 64	3 · 35–3 · 93	2527	15 · 32	14 · 76–15 · 88
Richer	1450	8 · 79	8 · 35–9 · 23	2058	12 · 48	11 · 96–12 · 99
Richest	2895	17 · 55	16 · 96–18 · 13	1062	6 · 44	6 · 06–6 · 82
Food-security status of households						
Food insecure	1500	9.09	8.66– 9.53	4207	25.51	24.84–26.17
Food secure	4261	25.84	25.17–26.50	6525	39.56	38.82–40.31
Patterns of contraceptive use						
Never users	851	5 · 16	4 · 82–5 · 50	1991	12 · 07	11 · 56–12 · 58
Current users	3473	21 · 06	20 · 44–21 · 69	6159	37 · 34	36 · 59–38 · 08
Past users	1437	8 · 71	8 · 27–9 · 15	2582	15 · 66	15 · 09–16 · 22
Menopausal status of women						
Not in menopause	4142	25 · 11	24 · 44–25 · 78	7307	44 · 30	43 · 55–45 · 06
In menopause	1619	9 · 82	9 · 35–10 · 28	3425	20 · 77	20 · 14–21 · 40
Number of living children						
0	532	3 · 23	2 · 95–3 · 50	789	4 · 78	4 · 45–5 · 12
1–2	3218	19 · 51	18 · 91–20 · 13	5199	31 · 52	30 · 80–32 · 23
3–4	1634	9 · 91	9 · 44–10 · 37	3542	21 · 48	2084–22 · 10
5+	377	2 · 29	2 · 05–2 · 52	1202	7 · 29	6 · 88–7 · 69
Educational status of women						
No education	1146	6 · 95	6 · 55–7 · 34	3311	20 · 08	19 · 45–20 · 70
Primary	1503	9 · 11	8 · 67–9 · 56	3436	20 · 83	20 · 20–21 · 45
Secondary	2241	13 · 59	13 · 05–14 · 12	3506	21 · 26	20 · 62–21 · 88
Higher	871	5 · 28	4 · 93–5 · 63	479	2 · 90	2 · 64–3 · 17
Age (years) of women at first birth						
<=13	190	1 · 15	0 · 99–1 · 32	365	2 · 21	1 · 98–2 · 44
13–17	2264	13 · 73	13 · 19–14 · 26	5021	30 · 44	29 · 73–31 · 16
18–22	2648	16 · 06	15 · 50–16 · 63	4650	28 · 19	27 · 51–28 · 89
> = 23	659	3 · 99	3 · 69–4 · 30	696	4 · 22	3 · 91–4 · 53
Watching TV at least once a week						
No	1507	9.14	8.70– 9.58	6783	41.13	40.38–41.88
Yes	4254	25.79	25.13–26.46	3949	23.94	23.29–24.59
Occupation of women						
Jobs that required manual labor	846	5 · 13	4 · 79–5 · 47	889	5 · 39	5 · 04–5 · 74
Jobs that required mostly sitting	335	2 · 03	1 · 81–2 · 25	438	2 · 66	2 · 41–2 · 91
Unemployed/housewives	4580	27 · 77	27 · 09–28 · 45	9405	57 · 02	56 · 27–57 · 78

**Fig. 2:**
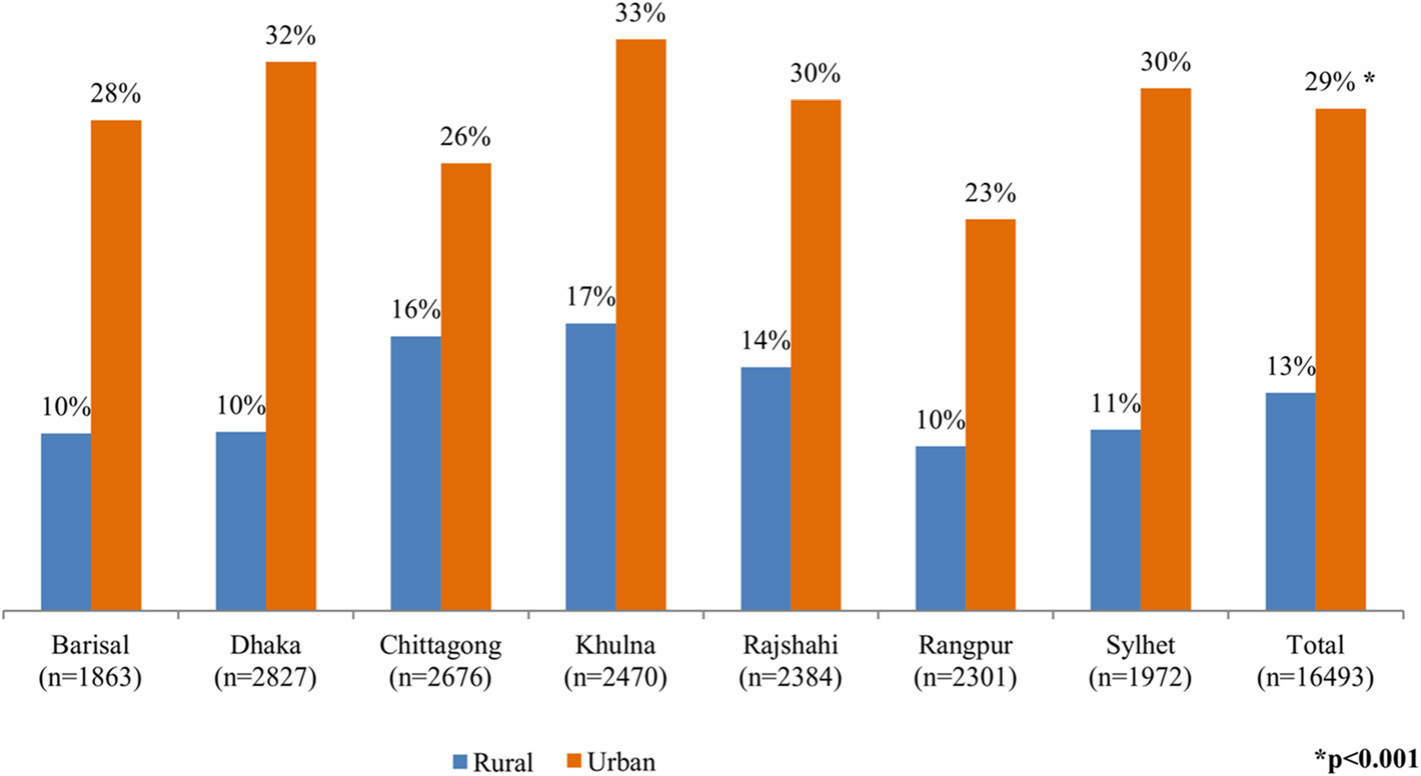
Prevalence of overweight or obesity among rural and urban women of different geographical divisions in Bangladesh

The prevalence of overweight and obesity was significantly higher in urban areas compared to rural areas (29 % vs. 13 %, *p* < 0 · 001) in all the geographic divisions. In urban areas of Dhaka and Khulna divisions had the highest prevalence of overweight and obesity compared to other divisions. However, in the case of rural areas, the prevalence of overweight and obesity was the highest in Chittagong, Khulna, and Rajshahi divisions compared to other divisions. The findings revealed that the prevalence of overweight and obesity was the lowest in Rangpur division compared to other divisions (Fig. 2).

Table 2 presents results of analyses of simple and multiple logistic regressions for rural women. Rural women of Khulna division had a higher risk of being overweight or obese compared to rural women of other divisions, from both regressions models. For example, rural women of Khulna division were 1 · 90 times (95 % CI 1 · 54-2 · 35, *p* < 0 · 001) more likely to be overweight or obese than rural women of Rangpur division and the odds ratio was 1.46 (95 % CI 1 · 13–1 · 78, *p* < 0 · 01) for rural women of Khulna division, after adjusting for other variables. The wealth index had a strong significant impact on the overweight or obesity status of rural women. The prevalence of overweight or obesity increased with the increase in household status of the wealth index. Rural women in food-secure households were 1.98 times (95 % CI 1 · 74–2 · 27, *p* < 0 · 001) more likely to become overweight or obese compared to those residing in food-insecure households, an impact diminished to 1 · 22 (95 % CI 1 · 05-1 · 42, *p* < 0 · 01) in the multivariable model. However, an increasing number of members showed a lower risk of being overweight or obese. Women with five or more living children were 0 · 76 times (95 % CI 0 · 57–1 · 01, *p* = 0 · 05) less likely to become overweight or obese than women who had no child, this association was strengthened by full adjustment (OR 0 · 59, 95 % CI 0 · 41–0 · 84, *p* < 0 · 01). Educational status also had a significant association with being overweight or obese. Women with higher education were 3 · 43 times (95 % CI 2 · 70–4 · 35, *p* < 0 · 001) more likely to become overweight or obese than non-educated rural women, this risk was 1.54 times more likely in the adjusted model. The risk of becoming overweight or obese increased with the increase in age. Women over 28 years were 3 · 87 times more likely to be overweight or obese than younger women. Widowed or divorced or separated rural women were less likely to be overweight or obese (OR 0.75, 95 % CI: 0.56–0.99, *p* < 0.05) compared to the married women. The risk of being overweight or obese was 1 · 31 times higher among the rural women who watched TV at least once a week compared to the women who did not watch TV at least once a week (Table 2).

**Table 2:** Odds ratio of logistic regression assessing the impacts of selected variables on overweight or obesity of rural women in Bangladesh

Variables with categories	Status of overweight or obesity of women					
	Simple logistic regression			Multiple logistic regression		
Division	OR	95 % CI	p value	OR	95 % CI	p value
Rangpur (Ref.)	1			1		
Dhaka	1 · 10	0 · 87–1 · 38	0 · 44	1 · 04	0 · 82–1 · 33	0 · 74
Chittagong	1 · 80	1 · 46–2 · 22	<0 · 001	1 · 42	1 · 13–1 · 78	<0 · 01
Khulna	1 · 90	1 · 54–2 · 35	<0 · 001	1 · 46	1 · 17–1 · 83	<0 · 01
Rajshahi	1 · 56	1 · 26–1 · 94	<0 · 001	1 · 35	1 · 07–1 · 70	<0 · 05
Barisal	1 · 09	0 · 85–1 · 39	0 · 50	1 · 02	0 · 78–1 · 33	0 · 89
Sylhet	1 · 11	0 · 88–1 · 41	0 · 38	0 · 93	0 · 72–1 · 21	0 · 59
No · of household members						
1–2 (Ref.)	1			1		
3–4	0 · 77	0 · 59–1 · 02	0 · 06	0 · 77	0 · 57–1 · 06	0 · 100
5+	0 · 72	0 · 56–0 · 94	<0 · 01	0 · 67	0 · 50–0 · 92	<0.05
Wealth index of households						
Poorest (Ref.)	1			1		
Poorer	1 · 35	1 · 07–1 · 71	<0 · 05	1 · 223	0 · 96–1 · 58	0 · 11
Middle	2 · 38	1 · 93–2 · 96	<0 · 001	1 · 86	1 · 46–2 · 37	<0 · 001
Richer	4 · 43	3 · 61–5 · 46	<0 · 001	3 · 01	2 · 34–3.89	<0 · 001
Richest	7 · 97	6 · 42–9 · 94	<0 · 001	4.75	3.57–6.33	<0 · 001
Food-security status of households						
Food insecure (Ref.)	1			1		
Food secure	1.98	1 · 74–2 · 27	<0 · 001	1 · 22	1 · 05–1 · 42	<0 · 01
Patterns of contraceptive use						
Never users (Ref.)	1			-		
Currently users	1 · 05	0 · 90–1 · 23	0 · 54	-	-	-
Past users	1 · 24	1 · 04–1 · 48	<0 · 05	-	-	-
Menopausal status of women						
Not in menopause (Ref.)	1			-		
In menopause	0 · 86	0 · 76–0 · 97	<0 · 05	-	-	-
Number of living children						
0 (Ref.)	1			1		
1–2	1 · 04	0 · 83–1 · 30	0 · 76	0 · 77	0 · 59– 0 · 99	<0 · 05
3–4	1 · 02	0 · 82–1 · 30	0 · 85	0 · 70	0 · 53–0 · 94	<0 · 05
5+	0 · 76	0 · 57–1 · 01	<0 · 05	0 · 59	0 · 41– 0 · 84	<0 · 01
Current marital status						
Married and living with husband (Ref.)	1			1		
Widowed/divorced/separated	0 · 79	0 · 61–1 · 02	0 · 07	0 · 75	0 · 56–0.99	<0.05
Educational status of women						
No education (Ref.)	1			1		
Primary	1 · 28	1 · 08–1 · 50	<0 · 01	1 · 17	0 · 98–1 · 40	0 · 09
Secondary	1 · 96	1 · 69–2 · 28	<0 · 001	1 · 44	1 · 19–1 · 76	<0 · 001
Higher	3 · 43	2 · 70–4 · 35	<0 · 001	1 · 54	1 · 15–2.06	<0 · 01
Age (years) of women						
18–22 (Ref.)	1			1		
23–27	2 · 06	1 · 66–2 · 57	<0 · 001	2 · 42	1 · 92–3 · 08	<0 · 001
28–32	2 · 85	2 · 29–3 · 55	<0 · 001	3 · 57	2 · 80– 4 · 58	<0 · 001
33–37	2 · 67	2 · 13–3 · 36	<0 · 001	3 · 71	2 · 84– 4 · 86	<0 · 001
38–42	2 · 61	2 · 08–3 · 29	<0 · 001	4.11	3.11– 5 · 46	<0 · 001
43+	2 · 51	2 · 00–3 · 16	<0 · 001	4.08	3.05– 5 · 47	<0 · 001
Age (years) of women at first birth						
<=13 (Ref.)	1			-		
13–17	1 · 24	0 · 87–1 · 81	0 · 25	-	-	-
18–22	1 · 55	1 · 10–2 · 27	0 · 02	-	-	-
> = 23	1 · 93	1 · 30–2 · 92	<0 · 01	-	-	-
Watching TV at least once a week						
No (Ref.)	1			1		
Yes	2 · 38	2.12–2 · 67	<0 · 001	1 · 31	1.14–1 · 51	<0 · 001
Occupation status of women						
Jobs that required manual labor (Ref.)	1			-		
Jobs that required mostly sitting	1 · 71	1 · 24–2 · 37	<0 · 01	-	-	-
Unemployed/housewives	1 · 18	0 · 95–1 · 47	0 · 15	-	-	-

Ref.: Reference category

Table 3 presents results of analyses of overweight and obesity-related data using simple and multiple logistic regressions for urban women. Urban women of Khulna division were at a higher risk of overweight or obesity than those of Rangpur division. The wealth index of households and food security had highly significant impacts on overweight or obesity of urban women. Women in the highest quintile household were 7 · 97 times (95 % CI 6.42–9.94, *p* < 0 · 001) more likely to be overweight or obese than women in the lowest quintile, which was 4.75 times more likely in the adjusted model. Women in the food-secure households were more likely to become overweight or obese compared to women in food-insecure households (OR 2 · 22, 95 % CI 1.92–2 · 59, *p* < 0 · 001). Highly-educated urban women were 2 · 93 times (95 % CI 2 · 41–3 · 57, *p* < 0 · 001) more likely to be overweight or obese compared to non-educated women. Urban women aged 38–42 years were more likely to be overweight or obese compared to other groups. Exposure of urban women to TV was an independent factor for overweight or obesity. In the full model, women who watched TV at least once a week were1.49 times (95 % CI 1.24–1.80, *p* < 0 · 001) more likely to be overweight or obese compared to women with no or less than once a week TV viewing. Unemployed women or housewives were at 1 · 85 times (95 % CI 1 · 55–2 · 23, *p* < 0 · 01) higher odds of being overweight or obese than women engaged in manual labor work, which had 1.44 times higher odds in the adjusted model (Table 3).

**Table 3:** Odds ratio of logistic regression assessing the impacts of selected variables on overweight or obesity of urban women in Bangladesh

Variables with categories	Status of overweight or obesity of women					
	Simple logistic regression			Multiple logistic regression		
Division	OR	95 % CI	p value	OR	95 % CI	p value
Rangpur (Ref.)	1			1		
Dhaka	1 · 59	1 · 28–1 · 98	<0 · 001	0 · 95	0 · 74–1 · 21	0 · 65
Chittagong	1 · 19	0 · 95–1 · 51	0 · 13	0 · 99	0 · 77–1 · 28	0 · 96
Khulna	1 · 69	1 · 34–2 · 13	<0 · 001	1 · 32	1 · 02–1 · 70	<0 · 05
Rajshahi	1 · 43	1 · 13–1 · 82	<0 · 01	1 · 09	0 · 84–1 · 41	0 · 52
Barisal	1 · 35	1 · 05–1 · 75	<0 · 05	1 · 07	0 · 81–1 · 42	0 · 62
Sylhet	1 · 46	1 · 14–1 · 88	<0 · 01	0 · 95	0 · 72–1 · 26	0 · 74
No · of household members						
1–2 (Ref.)	1			-		
3–4	1 · 20	0 · 91–1 · 61	0 · 21	-	-	-
5+	1 · 19	0 · 91–1 · 59	0 · 22	-	-	-
Wealth index of households						
Poorest (Ref.)	1			1		
Poorer	1 · 67	0 · 99–2 · 87	0 · 06	1 · 55	0 · 89–2 · 76	0 · 13
Middle	2 · 88	1 · 83–4 · 70	<0 · 001	2 · 45	1 · 51–4 · 15	<0 · 001
Richer	4 · 59	3 · 04–7 · 23	<0 · 001	3 · 32	2 · 11–5.49	<0 · 001
Richest	11 · 48	7 · 72–17 · 92	<0 · 001	6.36	4.02–10 · 55	<0 · 001
Food-security status of households						
Food insecure (Ref.)	1			1		
Food secure	2 · 22	1.92–2 · 59	<0 · 001	1 · 25	1 · 05–1 · 49	<0 · 01
Patterns of contraceptive use						
Never users (Ref.)	1			-		
Current users	1 · 11	0 · 94–1 · 32	0 · 21	-	-	-
Past users	1 · 12	0 · 93–1 · 35	0 · 25	-	-	-
Menopausal status of women						
Not in menopause (Ref.)	1			-		
In menopause	0 · 91	0 · 80–1 · 03	0 · 15	-	-	-
Number of living children						
0 (Ref.)	1			-		
1-2	1 · 49	1 · 20–1 · 87	<0 · 001	-	-	-
3-4	1 · 59	1 · 27–2 · 01	<0 · 001	–	-	-
5+	1 · 30	0 · 95–1 · 76	0 · 09	-	-	-
Current marital status						
Married and living with husband (Ref.)	1			-		
Widowed/divorced/separated	0 · 79	0 · 62–0 · 99	0 · 05	-	-	-
Educational status of women						
No education (Ref.)	1			1		
Primary	1 · 16	0 · 96–1 · 40	0 · 13	1 · 20	0 · 97–1 · 47	0 · 09
Secondary	1 · 87	1 · 58–2 · 21	<0 · 001	1 · 50	1 · 23–1 · 84	<0 · 001
Higher	2 · 93	2 · 41–3 · 57	<0 · 001	1 · 60	1 · 26–2.03	<0 · 001
Age (years) of women						
18–22 (Ref.)	1			1		
23–27	2 · 15	1 · 72–2 · 70	<0 · 001	2 · 12	1 · 69–2 · 68	<0 · 001
28–32	3 · 11	2 · 49–3 · 89	<0 · 001	2 · 97	2 · 35–3 · 76	<0 · 001
33–37	3 · 75	2 · 98–4 · 74	<0 · 001	3 · 70	2 · 90–4 · 74	<0 · 001
38–42	4 · 09	3 · 25–5 · 16	<0 · 001	4 · 56	3 · 57–5 · 86	<0 · 001
43+	3 · 66	2 · 92–4 · 61	<0 · 001	4.06	3.18–5.21	<0 · 001
Age (years) of women at first birth						
<=13 (Ref.)	1			-		
13–17	1 · 15	0 · 82–1 · 65	0 · 42	-	-	-
18–22	1 · 40	0 · 99–2 · 00	0 · 06	-	-	-
> = 23	2 · 25	1 · 56–3 · 29	<0 · 001	-	-	-
Watching TV at least once a week						
No	1			1		
Yes	3.01	2 · 58–3.53	<0 · 001	1 · 49	1 · 24–1 · 80	<0 · 001
Occupation status of women						
Jobs that required manual labor (Ref.)	1			1		
Jobs that required mostly sitting	2 · 33	1 · 75–3 · 08	<0 · 001	1 · 33	0 · 97–1 · 82	0 · 08
Unemployed/Housewife	1 · 85	1 · 55–2 · 23	<0 · 001	1 · 44	1 · 18–1 · 76	<0 · 001

Ref.: Reference category

Additionally, similar findings were found when we performed sensitivity analysis taking into consideration different cut-off points of BMI. When we considered a BMI cut-point of 23, the wealth index and food security status of households, age, educational status, and occupation of women were significantly associated with overweight and obesity of women. These indicators were also significantly associated with overweight and obesity when the BMI cut-point was 27 (data available in Additional file 1). We also analyzed data on overweight and obesity by excluding the underweight women (*n* = 3,680) from the dataset and we did not find any differences in outcomes from those that we observed in the full dataset analysis (data available in Additional file 1). Furthermore, we tested to understand whether there were any possible interactions among the variables such as: age and education; education and occupation, wealth index and food security. However, we did not find any significant interaction effect in the multiple logistic regressions and thus, we omitted them and fit the final model.

## Discussion

The findings of this analysis suggest that a large number of women in Bangladesh have been suffering from being overweight or obese. The results showed that a number of factors are associated with women becoming overweight or obese, varying according to place of residence, i.e. rural or urban. Thus, strategies to combat overweight and obesity among women should take into consideration the rural and urban contexts as the predictors of overweight and obesity influenced the women differently in both contexts. In multivariable analysis, geographical division, wealth index, food security, educational status, age, current marital status, watching TV, and occupational status had a significant association with the prevalence of overweight and obesity among rural and urban women of Bangladesh. In the context of geographical division, Khulna, located at the south-west of the country, is considered one of the high-risk divisions for both urban and rural women. However, according to the Nutrition Surveillance Project, the prevalence of other health indicators, including malnutrition and anemia in children and mothers, in Khulna division was lower than most other divisions in the country [11].

The findings of the present study further revealed that higher age of both rural and urban women was significantly associated with higher odds of women being overweight or obese compared to women in lower age-group. A previous study in Bangladesh has also demonstrated this association, with increased age as a significant predictor [8]. A similar trend was shown in another study in India that compared the data of the National Family Health Surveys of 1998 and 2005. In both the years, the association between overweight and obesity increased significantly with the increase in age [12]. Findings of a study also suggest that older age is significantly associated with considerable changes in body composition because fat-free mass decreases gradually and fat mass increases after 30 years of age [13, 14].

The results of the study showed that urban and rural women of wealthier households had higher odds of being overweight or obese compared to women of less-wealthy households. This finding is consistent with that of a study which assessed the association between socioeconomic status and BMI and overweight in low to middle-income countries and found that higher BMI and overweight were concentrated in higher socioeconomic groups [15]. Similar findings were revealed from a study in India; the increase in the prevalence of overweight was higher among the wealthiest women than among the poorest women [12]. Possible reasons for the positive association between increased wealth and being overweight are changes in dietary behavior with changes in income. Finding of a study suggest that, with the increase in income, the intake of higher energy and fat, and consumption of animal and processed foods increases, all of which are associated with overweight and obesity [16].

The findings of our study illustrate that the educational status of women has a positive and significant association with women being overweight or obese: higher educational status means a significantly increased odds of women being overweight or obese compared to women who have no education. The reasons behind this may be that higher-educated women are more likely to engage themselves in jobs that involve less physical movement, resulting in them becoming overweight or obese. This also correlated with a study that assessed how obesity varies by level of education, the results of which suggest that more-educated women in developing countries are more likely to be obese compared to women with less education [17]. On the contrary, a study in the north-west of Iran demonstrated that higher education of both men and women was negatively correlated with the status of their obesity, a pattern more consistent with that observed in high-income countries [18].

Exposure of women to television viewing had a significant association with their overweight or obesity status. This finding is consistent with the findings of other studies [19–21]. Previous literature has discussed television viewing as a proxy for both socioeconomic variables as well as for sitting time [22]. A study in a developed-country setting demonstrated that adults who watch television for more than two hours a day are more likely to be obese [20]. It is commonly observed that watching television is a usual leisure-time activity among women in Bangladesh, resulting in fewer calories being burned. More importantly, urban unemployed women and housewives were at a high risk of being overweight or obese due to the lack of physical labor, even though they had more access to formal education. Television viewing may also imply the ownership of television, making it a proxy for having higher wealth and better access to high caloric foods.

### Limitations

Our study has several limitations that need to be considered in future studies. Our analysis was based on secondary data, and the dataset lacked some important variables, such as food habits and physical activity of women, which we could have used in the models to fully understand the relationship between selected independent variables, and the overweight and obesity status of women. Additionally, previous study suggest that South Asian women have higher fat mass at the same levels of BMI as women from other parts of the world [23]. Hence, different susceptibilities at a given BMI are likely to increase risk of complications, such as diabetes or hypertension. The use of BMI of ≥25 kg/m^2^ as a cut-off point may not be ideal to predict the metabolic and cardiovascular risk profiles of women in this region; however, using different cut-off points does not appear to change any of the fundamental relationships [24, 25]. Moreover, smoking is known to have an inverse relationship with obesity and if the smoking rates are disproportionately distributed between wealth or education groups, this may confound the relationship seen between higher wealth and higher BMI. We were unable to consider smoking status of women in our analysis, as BDHS 2011 did not collect data on the smoking habits of women in Bangladesh. Furthermore, the analysis we did based on cross-sectional data also limits our ability to draw a causal conclusion. Additionally, our data are not representative of urban slum populations who are more likely to suffer from being underweight rather than overweight or obese. Despite these limitations, our results provide important contributions to the available data on the association between socioeconomic and demographic variables and the overweight and obesity status of women.

## Conclusions

Our analysis showed that a range of factors were responsible for the prevalence of overweight or obesity among reproductive-age women in Bangladesh. For example, geographical division, wealth index, food security of households, educational status, age, and television viewing, have a significant association with the prevalence of overweight and obesity. The rural and urban variation has a notable impact on overweight and obesity among women, not only in prevalence but also in the impact level from different risk factors. Therefore, interventions need to be tested rigorously to address the overweight and obesity epidemic among Bangladeshi women and to avert their spread as has already occurred among the poorer and rural populations of higher-income countries. The implementation of preventive interventions such as promoting higher level of physical activities, ensuring proper food policies and improving awareness through educational institutions and via campaigns within communities, may be helpful in impeding the increasing burden of overweight and obesity, especially among urban women. However, development and implementation of interventions based on these determinants is not an easy task. Experimental research is recommended to evaluate appropriate prevention strategies to reduce overweight and obesity among both rural and urban women of Bangladesh.

## Availability of data and materials

The data set of BDHS-2011 is available at the Demographic and Health Surveys Program. This is an open sources dataset, which is available on request at http://dhsprogram.com/what-we-do/survey/survey-display-349.cfm.

## Additional file

Additional file 1:
**Table S4**: Estimates of parameters of the multiple linear regression model for assessing the effects predictor variables on BMI. **Table S5**: Frequency and percentage distribution of overweight and obesity status among women and other selected variables (BMI cutoff 23). **Table S6**: Odds ratio of logistic regression assessing the effects of selected variables on overweight and obesity status of rural women (BMI cutoff 23). **Table S7**: Odds ratio of logistic regression assessing the effects of selected variables on overweight and obesity status of urban women (BMI cutoff 23). **Table S8**: Frequency and percentage distribution of overweight and obesity status among women and other selected variables (BMI cut off 27). **Table S9**: Odds ratio of logistic regression assessing the effects of selected variables on overweight and obesity status of rural women (BMI cut off 27). **Table S10**: Odds ratio of logistic regression assessing the effects of selected variables on overweight and obesity status of urban women (BMI cut off 27). **Table S11**: Odds ratio of logistic regression assessing the effects of selected variables on overweight and obesity status of urban women (excluded underweight women). **Table S12**: Odds ratio of logistic regression assessing the effects of selected variables on overweight and obesity status of rural women (excluded underweight women). **Table S13**: Odds ratio of logistic regression assessing the impacts of selected variables on obesity (BMI ≥ 30 kg/m^2^) of rural women in Bangladesh. **Table S14**: Odds ratio of logistic regression assessing the impacts of selected variables on obesity (BMI ≥ 30 kg/m^2^) of urban women in Bangladesh. **Table S15**: Generalized variance inflation (GVIF) values of final models of Rural and Urban women. (DOCX 93 kb)
