# Supplementary material for: Determinants of overweight or obesity among ever-married adult women in Bangladesh
Source: BMC Obes. 2016 Mar 1;3:13. doi: 10.1186/s40608-016-0093-5 (PMC4774107; doi:10.1186/s40608-016-0093-5)
Supplement: Additional file 1: — Table S4: Estimates of parameters of the multiple linear regression model for assessing the effects predictor variables on BMI. Table S5: Frequency and percentage distribution of overweight and obesity status among women and other selected variables (BMI cutoff 23). Table S6: Odds ratio of logistic regression assessing the effects of selected variables on overweight and obesity status of rural women (BMI cutoff 23). Table S7: Odds ratio of logistic regression assessing the effects of selected variables on overweight and obesity status of urban women (BMI cutoff 23). Table S8: Frequency and percentage distribution of overweight and obesity status among women and other selected variables (BMI cut off 27). Table S9: Odds ratio of logistic regression assessing the effects of selected variables on overweight and obesity status of rural women (BMI cut off 27). Table S10: Odds ratio of logistic regression assessing the effects of selected variables on overweight and obesity status of urban women (BMI cut off 27). Table S11: Odds ratio of logistic regression assessing the effects of selected variables on overweight and obesity status of urban women (excluded underweight women). Table S12: Odds ratio of logistic regression assessing the effects of selected variables on overweight and obesity status of rural women (excluded underweight women). Table S13: Odds ratio of logistic regression assessing the impacts of selected variables on obesity (BMI ≥ 30 kg/m2) of rural women in Bangladesh. Table S14: Odds ratio of logistic regression assessing the impacts of selected variables on obesity (BMI ≥ 30 kg/m2) of urban women in Bangladesh. Table S15: Generalized variance inflation (GVIF) values of final models of Rural and Urban women. (DOCX 93 kb) [file 40608_2016_93_MOESM1_ESM.docx]

**Supplementary File: Determinants of Overweight or Obesity among Ever-married Adult Women in Bangladesh**

**Table 4:** Estimates of parameters of the multiple linear regression model for assessing the effects predictor variables on BMI

| **Variables with categories** | **Estimate** | **Standard error** | **p-value** |
| --- | --- | --- | --- |
| **Divisions:** |  |  |  |
| Rangpur |  |  |  |
| Dhaka | -0·13 | 0·11 | 0·18 |
| Chittagong | 0·22 | 0·11 | 0·06 |
| Khulna | 0·38 | 0·11 | <0·001 |
| Rajshahi | 0·12 | 0·11 | 0·27 |
| Barisal | -0·04 | 0·12 | 0·51 |
| Sylhet | -0·45 | 0·12 | <0·001 |
| **Residence:**  Urban |  |  |  |
| Rural | -0·54 | 0·07 | <0·001 |
| **Wealth Index:** |  |  |  |
| Poorest |  |  |  |
| Poorer | 0·37 | 0·11 | <0·001 |
| Middle | 077 | 0·11 | <0·001 |
| Richer | 1·40 | 0·12 | <0·001 |
| Richest | 2.89 | 0·14 | <0·001 |
| **Food security status of households** |  |  |  |
| Food insecure |  |  |  |
| Food secure | 0·36 | 0·07 | <0·001 |
| **Menopausal status of women** |  |  |  |
| Not in menopause |  |  |  |
| In menopause | 0·12 | 0·07 | 0·11 |
| Current marital status: |  |  |  |
| Married and living with husband |  |  |  |
| Widowed/divorced/separated | -0·67 | 0·14 | <0·001 |
| **Occupation status of women** |  |  |  |
| Jobs that required manual labor |  |  |  |
| Jobs that required mostly sitting | 0·39 | 0·17 | <0·05 |
| Unemployed/Housewife | 0·37 | 0·10 | <0·001 |
| **Educational status of women** |  |  |  |
| No education |  |  |  |
| Primary | 0·26 | 0·09 | <0·01 |
| Secondary | 0·71 | 0·10 | <0·001 |
| Higher | 0·99 | 0·15 | <0·001 |
| **Watching TV at least once a week** |  |  |  |
| No |  |  |  |
| Yes | 0·50 | 0·08 | <0·001 |
| **Age of women** |  |  |  |
| 18-22 |  |  |  |
| 23-27 | 1·17 | 0·10 | <0·001 |
| 28-32 | 1·87 | 0·11 | <0·001 |
| 33-37 | 2·30 | 0·12 | <0·001 |
| 38-42 | 2·41 | 0·13 | <0·001 |
| 43+ | 2·23 | 0·14 | <0·001 |
| **Number of Household member** |  |  |  |
| 1-2 |  |  |  |
| 3-4 | -0·24 | 0·16 | 0·13 |
| 5+ | -0·40 | 0·16 | <0·05 |
| **Patterns of contraceptive use** |  |  |  |
| Never users |  |  |  |
| Currently users | 0·08 | 0·10 | 0·40 |
| Past users | 0·24 | 0·10 | <0·05 |
| **Age at first birth** |  |  |  |
| <=13 |  |  |  |
| 13-17 | -0·14 | 0·17 | 0·441 |
| 18-22 | -0·28 | 0·18 | 0·12 |
| >=23 | -0·40 | 0·21 | 0·06 |
| **Number of living children** |  |  |  |
| 0 |  |  |  |
| 1-2 | -0·15 | 0·13 | 0·25 |
| 3-4 | -0·47 | 0·16 | <0·01 |
| 5+ | -0·80 | 0·19 | <0·001 |

**Table 5:** Frequency and percentage distribution of overweight and obesity status among women and other selected variables (BMI cutoff 23)

| **Variables with categories** | **Frequency (n)** | **Percentage (%)** | **95% CI** |
| --- | --- | --- | --- |
| **Overweight and obesity status of women** |  |  |  |
| BMI < 23 | 11199 | 67·90 | 67·19- 68·61 |
| BMI>= 23 | 5294 | 32·09 | 31·39- 32·81 |
| **Age of women** |  |  |  |
| 18-22 | 3089 | 18·73 | 18·13-19·32 |
| 23-27 | 3445 | 20·89 | 20·27- 21·51 |
| 28-32 | 2903 | 17·60 | 17·02-18·18 |
| 33-37 | 2334 | 14·15 | 13·62- 14·68 |
| 38-42 | 2265 | 13·73 | 13·21-14·26 |
| 43+ | 2457 | 14·90 | 14·35-15·44 |
| **Current marital status** |  |  |  |
| Married and living with husband | 15425 | 93·52 | 93·15-93·90 |
| Widowed/divorced/separated | 1068 | 6·48 | 6·09-6·85 |
| **Place of residence** |  |  |  |
| Rural | 10732 | 65·07 | 64·34-65·79 |
| Urban | 5761 | 34·93 | 34·20-35·66 |
| **Division** |  |  |  |
| Barisal | 1863 | 11·30 | 10·81-11·78 |
| Chittagong | 2676 | 16·23 | 15·66-16·78 |
| Dhaka | 2827 | 17·14 | 16·57-17·72 |
| Khulna | 2470 | 14·98 | 14·43-15·52 |
| Rajshahi | 2384 | 14·45 | 13·92-14·99 |
| Rangpur | 2301 | 13·95 | 13·42-14·48 |
| Sylhet | 1972 | 11·95 | 11·46-12·45 |
| **Number of household member** |  |  |  |
| 1-2 | 726 | 4·40 | 4·09-4·71 |
| 3-4 | 5791 | 35·11 | 34·38-35·84 |
| 5+ | 9976 | 60·49 | 59·74-61·23 |
| **Wealth index of household** |  |  |  |
| Poorest | 2867 | 17·38 | 16·80-17·96 |
| Poorer | 3034 | 18·40 | 17·80-18·99 |
| Middle | 3127 | 18·96 | 18·36-19·56 |
| Richer | 3508 | 21·27 | 20·65-21·89 |
| Richest | 3957 | 23·99 | 23·34-24·64 |
| **Food security status of households** |  |  |  |
| Food insecure | 5100 | 32·10 | 31.39-32.82 |
| Food secure | 10786 | 67·90 | 67.18-, 68.61 |
| **Patterns of contraceptive use** |  |  |  |
| Never users | 2842 | 17·23 | 16·66-17·81 |
| Currently users | 9632 | 58·40 | 57·65-59·15 |
| Past users | 4019 | 24·37 | 23·71-25·02 |
| **Menopausal status of women** |  |  |  |
| Not in menopause | 11449 | 69·42 | 68·7-70·12 |
| In menopause | 5044 | 30·58 | 29·88-31·29 |
| **Number of living children** |  |  |  |
| 0 | 1321 | 8·01 | 7·60-8·43 |
| 1-2 | 8417 | 51·03 | 50·26-51·79 |
| 3-4 | 5176 | 31·38 | 30·67-32·09 |
| 5+ | 1579 | 9·57 | 9·12-10·02 |
| **Educational status of women** |  |  |  |
| No education | 4457 | 27·02 | 26·35-27·70 |
| Primary | 4939 | 29·95 | 29·25-30·65 |
| Secondary | 5747 | 34·85 | 34·12-35·57 |
| Higher | 1350 | 8·18 | 7·77-8·60 |
| **Age of women at first birth** |  |  |  |
| <=13 | 555 | 3·37 | 3·09-3·64 |
| 13-17 | 7285 | 44·17 | 43·41-44·93 |
| 18-22 | 7298 | 44·25 | 43·49-45·01 |
| >=23 | 1355 | 8·21 | 7·80-8·63 |
| **Watching TV at least once a week** |  |  |  |
| No | 8290 | 50.26 | 49.50-51.03 |
| Yes | 8203 | 49.74 | 48.97-50.50 |
| **Occupation status of women** |  |  |  |
| Jobs that required manual labor | 1735 | 10·52 | 10·05-10·99 |
| Jobs that required mostly sitting | 773 | 4·69 | 4·36-5·01 |
| Unemployed/Housewife | 13985 | 84·79 | 84·24-85·34 |

**Table 6:** Odds ratio of logistic regression assessing the effects of selected variables on overweight and obesity status of rural women (BMI cutoff 23)

| **Variables with categories** | **Status of overweight and obesity of women** | | | | | |
| --- | --- | --- | --- | --- | --- | --- |
|  | Simple Logistic Regression | | | Multiple logistic Regression | | |
| **Divisions** | **OR** | **95% CI** | **p-value** | **OR** | **95% CI** | **p-value** |
| Rangpur | 1 |  |  | 1 |  |  |
| Dhaka | 1·03 | 0·86-1·22 | 0·77 | 0·96 | 0·80-1·16 | 0·70 |
| Chittagong | 1·81 | 1·54-2·12 | <0·01 | 1·49 | 1·25-1·77 | <0·001 |
| Khulna | 1·90 | 1·62-2·23 | <0·01 | 1·50 | 1·26-1·78 | <0·001 |
| Rajshahi | 1·54 | 1·30-1·81 | <0·01 | 1·31 | 1·10-1·56 | <0·01 |
| Barisal | 1·11 | 0·93-1·33 | 0·24 | 1·04 | 0·85- 1·27 | 0·70 |
| Sylhet | 0·97 | 0·81-1·17 | 0·77 | 0·82 | 0·67- 1·01 | 0·06 |
| **Number of household member** |  |  |  |  |  |  |
| 1-2 | 1 |  |  | 1 |  |  |
| 3-4 | 0·93 | 0·75-1·16 | 0·51 | 0·87 | 0·68- 1·32 | 0·30 |
| 5+ | 0·87 | 0·71-1·09 | 0·22 | 0·80 | 0·62- 1·03 | 0·08 |
| **Wealth index of household** |  |  |  |  |  |  |
| Poorest | 1 |  |  | 1 |  |  |
| Poorer | 1·59 | 1·36-1·87 | <0·001 | 1·48 | 1·24- 1·76 | <0·001 |
| Middle | 2·64 | 2·27-3·08 | <0·001 | 2·15 | 1·81-1.81 | <0·001 |
| Richer | 4·13 | 3·55- 4·81 | <0·001 | 3·00 | 2·49- 3·63 | <0·001 |
| Richest | 7·91 | 6·66-9·41 | <0·001 | 5·19 | 4·16-6.49 | <0·001 |
| **Food security status of households** |  |  |  |  |  |  |
| Food insecure | 1 |  |  | 1 |  |  |
| Food secure | 1·78 | 1·61-1·96 | <0·001 | 1·15 | 1·03- 1·29 | <0·05 |
| **Patterns of contraceptive use** |  |  |  |  |  |  |
| Never users | 1 |  |  | 1 |  |  |
| Currently users | 1·13 | 1·01-1·28 | <0·05 | 1·01 | 0·86- 1·17 | 0·62 |
| Past users | 1·39 | 1·21-1·59 | <0·001 | 1·24 | 1·06- 1·45 | <0·01 |
| **Menopausal status of women** |  |  |  |  |  |  |
| Not in menopause | 1 |  |  | - |  |  |
| In menopause | 0·96 | 0·87-1·05 | 0·35 | - | - | - |
| **Number of living children** |  |  |  |  |  |  |
| 0 | 1 |  |  | 1 |  |  |
| 1-2 | 1·23 | 1·03-1·47 | <0.05 | 0·95 | 0·77-1·17 | 0·62 |
| 3-4 | 1·13 | 0·95-1·37 | 0·18 | 0·76 | 0·60-0·97 | <0.05 |
| 5+ | 0·89 | 0·72-1·11 | 0·32 | 0·67 | 0·49-0·87 | <0·01 |
| **Current marital status** |  |  |  |  |  |  |
| Married and living with husband | 1 |  |  | 1 |  |  |
| Widowed/divorced/separated | 0·74 | 0·61-0·90 | <0·01 | 0·67 | 0·53-0·84 | <0·001 |
| **Educational status of women** |  |  |  |  |  |  |
| No education | 1 |  |  | 1 |  |  |
| Primary | 1·24 | 1·10- 1·39 | <0·001 | 1·11 | 0·97-1·27 | 0·13 |
| Secondary | 1·76 | 1·57- 1·97 | <0·001 | 1·25 | 1·08-1·45 | <0·01 |
| Higher | 3·17 | 2·59- 3·87 | <0·001 | 1·41 | 1·10-1·79 | <0·01 |
| **Age of women** |  |  |  |  |  |  |
| 18-22 | 1 |  |  | 1 |  |  |
| 23-27 | 1·85 | 1·58- 2·16 | <0·001 | 2·13 | 1·79-2·52 | <0·001 |
| 28-32 | 2·42 | 2·07- 2·84 | <0·001 | 3·07 | 2·56-3·69 | <0·001 |
| 33-37 | 2·34 | 1·98- 2·76 | <0·001 | 3·32 | 2·72-4·06 | <0·001 |
| 38-42 | 2·26 | 1·92- 2·68 | <0·001 | 3·52 | 2·85-4·35 | <0·001 |
| 43+ | 2·13 | 1·80-2·51 | <0·001 | 3·29 | 2·64-4·11 | <0·001 |
| **Age of women at first birth** |  |  |  |  |  |  |
| <=13 | 1 |  |  | - |  |  |
| 13-17 | 1·05 | 0·82- 1·37 | 0·69 |  |  |  |
| 18-22 | 1·23 | 0·95- 1·59 | 0·12 |  |  |  |
| >=23 | 1·58 | 1·18- 2·13 | <0·01 |  |  |  |
| **Watching TV at least once a week** |  |  |  |  |  |  |
| No | 1 |  |  |  |  |  |
| Yes | 2·13 | 1·94- 2·32 | <0·001 | 1·24 | 1.11-1·38 | <0.001 |
| **Occupation status of women** |  |  |  |  |  |  |
| Jobs that required manual labor | 1 |  |  |  |  |  |
| Jobs that required mostly sitting | 1·68 | 1·30- 2·17 | <0·001 |  |  |  |
| Unemployed/Housewife | 1·19 | 1·01- 1·41 | <0.05 |  |  |  |

**Table 7:** Odds ratio of logistic regression assessing the effects of selected variables on overweight and obesity status of urban women (BMI cutoff 23)

| **Variables with categories** | **Status of overweight and obesity of women** | | | | | |
| --- | --- | --- | --- | --- | --- | --- |
|  | **Simple Logistic Regression** | | | **Multiple logistic Regression** | | |
| **Divisions** | **OR** | **95% CI** | **p-value** | **OR** | **95% CI** | **p-value** |
| Rangpur | 1 |  |  | - |  |  |
| Dhaka | 1·55 | 1·28- 1·88 | <0·001 | - | - | - |
| Chittagong | 1·12 | 0·92- 1·37 | 0·26 | - | - | - |
| Khulna | 1·42 | 1·16- 1·75 | <0·01 | - | - | - |
| Rajshahi | 1·32 | 1·07- 1·62 | <0·05 | - | - | - |
| Barisal | 1·37 | 1·09- 1·71 | <0·01 | - | - | - |
| Sylhet | 1·30 | 1·04- 1·63 | <0·05 | - | - | - |
| **Number of household member** |  |  |  |  |  |  |
| 1-2 | 1 |  |  | 1 |  |  |
| 3-4 | 1·24 | 0·96- 1·61 | 0·09 | 0.94 | 0.75-1.26 | 0.67 |
| 5+ | 1·21 | 0·95- 1·56 | 0·13 | 0.83 | 0.63-1.11 | 0.21- |
| **Wealth index of household** |  |  |  |  |  |  |
| Poorest | 1 |  |  | 1 |  |  |
| Poorer | 1·90 | 1·32-2·77 | <0·01 | 1·74 | 1·17- 2·62 | <0·01 |
| Middle | 2·76 | 1·98-3·89 | <0·001 | 2·31 | 1·61-3·67 | <0·001 |
| Richer | 4·32 | 3·21-5·94 | <0·001 | 3·24 | 2·32-4.62 | <0·001 |
| Richest | 10·32 | 7·73-14·05 | <0·001 | 5.72 | 4·07-8.20 | <0·001 |
| **Food security status of households** |  |  |  |  |  |  |
| Food insecure | 1 |  |  | 1 |  |  |
| Food secure | 2·20 | 1·93-2·50 | <0·001 | 1·25 | 1·08-1·45 | <0·01 |
| **Patterns of contraceptive use** |  |  |  |  |  |  |
| Never users | 1 |  |  | - |  |  |
| Currently users | 1·16 | 1·00- 1·35 | 0·06 | - | - | - |
| Past users | 1·17 | 0·99-1·39 | 0·07 | - | - | - |
| **Menopausal status of women** |  |  |  |  |  |  |
| Not in menopause | 1 |  |  | - |  |  |
| In menopause | 0·87 | 0·78- 0·98 | <0·05 | - | - | - |
| **Number of living children** |  |  |  |  |  |  |
| 0 | 1 |  |  | - |  |  |
| 1-2 | 1·42 | 1·18-1·72 | <0·001 | - | - | - |
| 3-4 | 1·45 | 1·18- 1·77 | <0·001 | - | - | - |
| 5+ | 1·30 | 0·99- 1·70 | 0·06 | - | - | - |
| **Current marital status** |  |  |  |  |  |  |
| Married and living with husband | 1 |  |  | - |  |  |
| Widowed/divorced/separated | 0·78 | 0·63-0·95 | <0·05 | - | - | - |
| **Educational status of women** |  |  |  |  |  |  |
| No education | 1 |  |  | 1 |  |  |
| Primary | 1·08 | 0·92-1·26 | 0·36 | 1·09 | 0·91- 1·31 | 0·33 |
| Secondary | 1·82 | 1·58-2·12 | <0·001 | 1·56 | 1·30-1·87 | <0·001 |
| Higher | 3·13 | 2·61-3·77 | <0·001 | 1·84 | 1·45- 2·35 | <0·001 |
| **Age of women** |  |  |  |  |  |  |
| 18-22 | 1 |  |  | 1 |  |  |
| 23-27 | 2·01 | 1·67- 2·41 | <0·001 | 2·05 | 1·69-2·49 | <0·001 |
| 28-32 | 3·04 | 2·53-3·67 | <0·001 | 3·10 | 2·54-3·80 | <0·001 |
| 33-37 | 3·95 | 3·24-4·82 | <0·001 | 4·40 | 3·53-5·49 | <0·001 |
| 38-42 | 3·21 | 2·64-3·91 | <0·001 | 3·89 | 3·13-4·86 | <0·001 |
| 43+ | 3·05 | 2·52- 3·70 | <0·001 | 3·75 | 3.01-4·67 | <0·001 |
| **Age of women at first birth** |  |  |  |  |  |  |
| <=13 | 1 |  |  | 1 |  |  |
| 13-17 | 0·92 | 0·68-1·24 | 0·59 | 0·78 | 0·56-1·09 | 0·15 |
| 18-22 | 1·06 | 0·79-1·43 | 0·70 | 0·73 | 0·52-1·02 | 0·06 |
| >=23 | 1·76 | 1·27-2·44 | <0·01 | 0·63 | 0·43-0·92 | <0·05 |
| **Watching TV at least once a week** |  |  |  |  |  |  |
| No | 1 |  |  | 1 |  |  |
| Yes | 2·82 | 2·48-3·20 | <0·001 | 1·44 | 1·23-1·69 | <0·001 |
| **Occupation status of women** |  |  |  |  |  |  |
| Jobs that required manual labor | 1 |  |  | 1 |  |  |
| Jobs that required mostly sitting | 2·45 | 1·90-3·18 | <0·001 | 1·52 | 1·13-2.04 | <0·01 |
| Unemployed/Housewife | 1·78 | 1·53-2·08 | <0·001 | 1·48 | 1·25-1·77 | <0.001 |

**Table 8:** Frequency and percentage distribution of overweight and obesity status among women and other selected variables (BMI cut off 27)

| **Variables with categories** | **Frequency (n)** | **Percentage (%)** | **95% CI** |
| --- | --- | --- | --- |
| **Overweight and obesity status of women** |  |  |  |
| BMI < 27 | 14848 | 90·03 | 89·57- 90·48 |
| BMI>= 27 | 1645 | 9·97 | 9·52- 10·43 |
| **Age of women** |  |  |  |
| 18-22 | 3089 | 18·73 | 18·13-19·32 |
| 23-27 | 3445 | 20·89 | 20·27- 21·51 |
| 28-32 | 2903 | 17·60 | 17·02-18·18 |
| 33-37 | 2334 | 14·15 | 13·62- 14·68 |
| 38-42 | 2265 | 13·73 | 13·21-14·26 |
| 43+ | 2457 | 14·90 | 14·35-15·44 |
| **Current marital status** |  |  |  |
| Married and living with husband | 15425 | 93·52 | 93·15-93·90 |
| Widowed/divorced/separated | 1068 | 6·48 | 6·09-6·85 |
| **Place of residence** |  |  |  |
| Rural | 10732 | 65·07 | 64·34-65·79 |
| Urban | 5761 | 34·93 | 34·20-35·66 |
| **Division** |  |  |  |
| Barisal | 1863 | 11·30 | 10·81-11·78 |
| Chittagong | 2676 | 16·23 | 15·66-16·78 |
| Dhaka | 2827 | 17·14 | 16·57-17·72 |
| Khulna | 2470 | 14·98 | 14·43-15·52 |
| Rajshahi | 2384 | 14·45 | 13·92-14·99 |
| Rangpur | 2301 | 13·95 | 13·42-14·48 |
| Sylhet | 1972 | 11·95 | 11·46-12·45 |
| **Number of household member** |  |  |  |
| 1-2 | 726 | 4·40 | 4·09-4·71 |
| 3-4 | 5791 | 35·11 | 34·38-35·84 |
| 5+ | 9976 | 60·49 | 59·74-61·23 |
| **Wealth index of household** |  |  |  |
| Poorest | 2867 | 17·38 | 16·80-17·96 |
| Poorer | 3034 | 18·40 | 17·80-18·99 |
| Middle | 3127 | 18·96 | 18·36-19·56 |
| Richer | 3508 | 21·27 | 20·65-21·89 |
| Richest | 3957 | 23·99 | 23·34-24·64 |
| **Food security status of households** |  |  |  |
| Food insecure | 5100 | 32·10 | 31.39-32.82 |
| Food secure | 10786 | 67·90 | 67.18-, 68.61 |
| **Patterns of contraceptive use** |  |  |  |
| Never users | 2842 | 17·23 | 16·66-17·81 |
| Currently users | 9632 | 58·40 | 57·65-59·15 |
| Past users | 4019 | 24·37 | 23·71-25·02 |
| **Menopausal status of women** |  |  |  |
| Not in menopause | 11449 | 69·42 | 68·7-70·12 |
| In menopause | 5044 | 30·58 | 29·88-31·29 |
| **Number of living children** |  |  |  |
| 0 | 1321 | 8·01 | 7·60-8·43 |
| 1-2 | 8417 | 51·03 | 50·26-51·79 |
| 3-4 | 5176 | 31·38 | 30·67-32·09 |
| 5+ | 1579 | 9·57 | 9·12-10·02 |
| **Educational status of women** |  |  |  |
| No education | 4457 | 27·02 | 26·35-27·70 |
| Primary | 4939 | 29·95 | 29·25-30·65 |
| Secondary | 5747 | 34·85 | 34·12-35·57 |
| Higher | 1350 | 8·18 | 7·77-8·60 |
| **Age of women at first birth** |  |  |  |
| <=13 | 555 | 3·37 | 3·09-3·64 |
| 13-17 | 7285 | 44·17 | 43·41-44·93 |
| 18-22 | 7298 | 44·25 | 43·49-45·01 |
| >=23 | 1355 | 8·21 | 7·80-8·63 |
| **Watching TV at least once a week** |  |  |  |
| No | 8290 | 50.26 | 49.50-51.03 |
| Yes | 8203 | 49.74 | 48.97-50.50 |
| **Occupation status of women** |  |  |  |
| Jobs that required manual labor | 1735 | 10·52 | 10·05-10·99 |
| Jobs that required mostly sitting | 773 | 4·69 | 4·36-5·01 |
| Unemployed/Housewife | 13985 | 84·79 | 84·24-85·34 |

**Table 9:** Odds ratio of logistic regression assessing the effects of selected variables on overweight and obesity status of rural women ( BMI cut off 27)

| **Variables with categories** | **Status of overweight and obesity of women** | | | | | |
| --- | --- | --- | --- | --- | --- | --- |
|  | **Simple Logistic Regression** | | | **Multiple logistic Regression** | | |
| **Divisions** | **OR** | **95% CI** | **p-value** | **OR** | **95% CI** | **p-value** |
| Rangpur | 1 |  |  | 1 |  |  |
| Dhaka | 1·23 | 0·88-1·72 | 0·22 | 1·18 | 0·84-1·68 | 0·34 |
| Chittagong | 2·14 | 1·59-2·90 | <0·001 | 1·62 | 1·19-2·24 | <0·01 |
| Khulna | 2·10 | 1·56-2·85 | <0·001 | 1·60 | 1·17-2·21 | <0·01 |
| Rajshahi | 1·66 | 1·21-2·28 | <0·01 | 1·46 | 1·06-2.04 | <0·05 |
| Barisal | 1·14 | 0·80-1·64 | 0·46 | 1·07 | 0·73-1·56 | <0·05 |
| Sylhet | 1·36 | 0·97-1·92 | 0·07 | 1·09 | 0·77-1·57 | 0·62 |
| **Number of household member** |  |  |  |  |  |  |
| 1-2 | 1 |  |  | 1 |  |  |
| 3-4 | 0·74 | 0·53-1·08 | 0·10 | 0·69 | 0·48-1·03 | 0·06 |
| 5+ | 0·66 | 0·48-0·95 | <0·05 | 0·54 | 0·38-0·79 | <0.01 |
| **Wealth index of household** |  |  |  |  |  |  |
| Poorest | 1 |  |  | 1 |  |  |
| Poorer | 1·22 | 0·86-1·74 | 0·27 | 1·05 | 0·72-1·52 | 0·82 |
| Middle | 2·43 | 1·79-3·35 | <0·001 | 1·77 | 1·26-2·52 | <0·01 |
| Richer | 4·38 | 3·26-5·97 | <0·001 | 2.77 | 1.95-4·00 | <0·001 |
| Richest | 9·29 | 6·89-12·71 | <0·001 | 5.23 | 3.56-7.77 | <0·001 |
| **Food security status of households** |  |  |  |  |  |  |
| Food insecure | 1 |  |  | 1 |  |  |
| Food secure | 2·34 | 1·93-2·85 | <0·001 | 1·44 | 1·16-1·79 | <0·01 |
| **Patterns of contraceptive use** |  |  |  |  |  |  |
| Never users | 1 |  |  | - |  |  |
| Currently users | 0·91 | 0·74-1·13 | 0·38 | - | - | - |
| Past users | 1·021 | 0·81-1·30 | 0·86 | - | - | - |
| **Menopausal status of women** |  |  |  |  |  |  |
| Not in menopause | 1 |  |  | - |  |  |
| In menopause | 0·86 | 0·72-1·02 | 0·08 | - | - | - |
| **Number of living children** |  |  |  |  |  |  |
| 0 | 1 |  |  | - |  |  |
| 1-2 | 1·00 | 0·74-1·38 | 0·99 | - | - | - |
| 3-4 | 1·00 | 0·74-1·40 | 0·98 | - | - | - |
| 5+ | 0·82 | 0·56-1·21 | 0·32 | - | - | - |
| **Current marital status** |  |  |  |  |  |  |
| Married and living with husband | 1 |  |  | - |  |  |
| Widowed/divorced/separated | 0·86 | 0·60-1·20 | 0·40 | - | - | - |
| **Educational status of women** |  |  |  |  |  |  |
| No education | 1 |  |  | 1 |  |  |
| Primary | 1·18 | 0·94-1·48 | 0·15 | 1·10 | 0·86-1·40 | 0·47 |
| Secondary | 1·93 | 1·57-2·38 | <0·01 | 1·39 | 1·07-1·82 | <0·05 |
| Higher | 2·99 | 2·15-4·11 | <0·01 | 1·33 | 0.90-1·94 | 0·15 |
| **Age of women** |  |  |  |  |  |  |
| 18-22 | 1 |  |  | 1 |  |  |
| 23-27 | 2·10 | 1·52-2·93 | <0·001 | 2·22 | 1·60-3·13 | <0·001 |
| 28-32 | 2·85 | 2·07-3·96 | <0·001 | 3·11 | 2·24-4·37 | <0·001 |
| 33-37 | 3·44 | 2·50-4·79 | <0·001 | 3.95 | 2.82-5.61 | <0·001 |
| 38-42 | 2·91 | 2·09-4·10 | <0·001 | 3.62 | 2·53-5·22 | <0·001 |
| 43+ | 2·95 | 2·13-4·13 | <0·001 | 3.66 | 2·56-5.29 | <0·001 |
| **Age of women at first birth** |  |  |  |  |  |  |
| <=13 | 1 |  |  | - |  |  |
| 13-17 | 1·16 | 0·72-1·99 | 0·57 | - | - | - |
| 18-22 | 1·45 | 0·91-2·49 | 0·14 | - | - | - |
| >=23 | 1·97 | 1·16-3·52 | <0·05 | - | - | - |
| **Watching TV at least once a week** |  |  |  |  |  |  |
| No | 1 |  |  | 1 |  |  |
| Yes | 2·45 | 2.09-2·88 | <0·001 | 1·25 | 1.03-1·52 | <0.05 |
| **Occupation status of women** |  |  |  |  |  |  |
| Jobs that required manual labor | 1 |  |  | - |  |  |
| Jobs that required mostly sitting | 1·87 | 1·19-2·94 | <0·01 | - | - | - |
| Unemployed/Housewife | 1·28 | 0·94-1·78 | 0·13 | - | - | - |

**Table 10:** Odds ratio of logistic regression assessing the effects of selected variables on overweight and obesity status of urban women (BMI cut off 27)

| **Variables with categories** | **Status of overweight and obesity of women** | | | | | |
| --- | --- | --- | --- | --- | --- | --- |
|  | **Simple Logistic Regression** | | | **Multiple logistic Regression** | | |
| **Divisions** | **OR** | **95% CI** | **p-value** | **OR** | **95% CI** | **p-value** |
| Rangpur | 1 |  |  | - |  |  |
| Dhaka | 1·80 | 1·38- 2·37 | <0·001 | - | - | - |
| Chittagong | 1·35 | 1·02- 1·81 | <0·05 | - | - | - |
| Khulna | 1·53 | 1·15- 2·06 | <0·01 | - | - | - |
| Rajshahi | 1·32 | 0·98- 1·79 | 0·07 | - | - | - |
| Barisal | 1·43 | 1·05- 1·97 | <0·05 | - | - | - |
| Sylhet | 1·53 | 1·12- 2·08 | <0·01 | - | - | - |
| **Number of household member** |  |  |  |  |  |  |
| 1-2 | 1 |  |  | - |  |  |
| 3-4 | 1·20 | 0·85- 1·74 | 0·31 | - | - | - |
| 5+ | 1·26 | 0·90- 1·80 | 0·20 | - | - | - |
| **Wealth index of household** |  |  |  |  |  |  |
| Poorest | 1 |  |  | 1 |  |  |
| Poorer | 2·24 | 0·88-6·43 | 0·11 | 2.19 | 0·81-6.90 | 0·18 |
| Middle | 5·34 | 2·43-14·08 | <0·001 | 4·62 | 1.98-13·50 | <0·01 |
| Richer | 9·08 | 4·36-23·23 | <0·001 | 6.47 | 3·52-18·94 | <0·001 |
| Richest | 23·81 | 11·6-60·38 | <0·001 | 12.40 | 5.53-35·44 | <0·001 |
| **Food security status of households** |  |  |  |  |  |  |
| Food insecure | 1 |  |  | 1 |  |  |
| Food secure | 2·53 | 2·08- 3·09 | <0·001 | 1·36 | 1·10- 1·70 | <0·01 |
| **Patterns of contraceptive use** |  |  |  |  |  |  |
| Never users | 1 |  |  | - |  |  |
| Currently users | 1·07 | 0·87-1·31 | 0·53 | - | - | - |
| Past users | 1·09 | 0·87- 1·37 | 0·46 | - | - | - |
| **Menopausal status of women** |  |  |  |  |  |  |
| Not in menopause | 1 |  |  | - |  |  |
| In menopause | 0·93 | 0·79- 1·08 | 0·34 | - | - | - |
| **Number of living children** |  |  |  |  |  |  |
| 0 | 1 |  |  | - |  |  |
| 1-2 | 1·43 | 1·09- 1·89 | <0·05 | - | - | - |
| 3-4 | 1·64 | 1·25- 2·20 | <0·01 | - | - | - |
| 5+ | 1·34 | 0·92- 1·95 | 0·13 | - | - | - |
| **Current marital status** |  |  |  |  |  |  |
| Married and living with husband | 1 |  |  | - |  |  |
| Widowed/divorced/separated | 0·78 | 0·58- 1·04 | 0·10 | - | - | - |
| **Educational status of women** |  |  |  |  |  |  |
| No education | 1 |  |  | 1 |  |  |
| Primary | 1·11 | 0·88-1·41 | 0·39 | 1·11 | 0·86- 1·44 | 0·40 |
| Secondary | 1·96 | 1·60-2·43 | <0·001 | 1·55 | 1·22- 1·97 | <0·001 |
| Higher | 2·69 | 2·12- 3·42 | <0·001 | 1·50 | 1.13- 1·99 | <0.01 |
| **Age of women** |  |  |  |  |  |  |
| 18-22 | 1 |  |  | 1 |  |  |
| 23-27 | 1·91 | 1·43- 2·57 | <0·001 | 1·86 | 1·38-2·50 | <0·001 |
| 28-32 | 3·03 | 2·29-4·06 | <0·001 | 2·84 | 2·12-3·83 | <0·001 |
| 33-37 | 3·56 | 2·66- 4·79 | <0·001 | 3·48 | 2·58-4·75 | <0·001 |
| 38-42 | 4·09 | 3·08-5·49 | <0·001 | 4·51 | 3·35- 6.14 | <0·001 |
| 43+ | 4·03 | 3·05-5·40 | <0·001 | 4·45 | 3·31-6.05 | <0·001 |
| **Age of women at first birth** |  |  |  |  |  |  |
| <=13 | 1 |  |  | - |  |  |
| 13-17 | 0·93 | 0·63- 1·42 | 0·73 | - | - | - |
| 18-22 | 1·11 | 0·75- 1·69 | 0·61 | - | - | - |
| >=23 | 1·82 | 1·21- 2·84 | <0·01 | - | - | - |
| **Watching TV at least once a week** |  |  |  |  |  |  |
| No | 1 |  |  | 1 |  |  |
| Yes | 3.20 | 2·62- 3·95 | <0·001 | 1·47 | 1·16- 1·86 | <0·01 |
| **Occupation status of women** |  |  |  |  |  |  |
| Jobs that required manual labor | 1 |  |  | 1 |  |  |
| Jobs that required mostly sitting | 1·96 | 1·37- 2·78 | <0·001 | 1·09 | 0·74-1·60 | 0·67 |
| Unemployed/Housewife | 1·93 | 1·54- 2·46 | <0·001 | 1·45 | 1·14-1·87 | <0·01 |

**Table 11:** Odds ratio of logistic regression assessing the effects of selected variables on overweight and obesity status of urban women (excluded underweight women)

| **Variables with categories** | **Status of overweight and obesity of women** | | | | | |
| --- | --- | --- | --- | --- | --- | --- |
|  | **Simple logistic regression** | | | **Multiple logistic regression** | | |
| **Division** | **OR** | **95% CI** | **p-value** | **OR** | **95% CI** | **p-value** |
| Rangpur | 1 |  |  | 1 |  |  |
| Dhaka | 1·41 | 1·13-1·77 | <0·01 | 0·91 | 0·72-1·17 | 0·48 |
| Chittagong | 1·16 | 0·92-1·48 | 0·22 | 0·99 | 0·77-1·30 | 0·99 |
| Khulna | 1·62 | 1·28-2·06 | <0·001 | 1·33 | 1·02-1·72 | <0.05 |
| Rajshahi | 1·36 | 1·07-1·75 | <0·05 | 1·09 | 0·84-1·43 | 0·51 |
| Barisal | 1·31 | 1·00-1·70 | <0·05 | 1·09 | 0·82-1·45 | 0·64 |
| Sylhet | 1·51 | 1·16-1·96 | <0·01 | 1.00 | 0·75-1·33 | 0·81 |
| **Number of household members** |  |  |  |  |  |  |
| 1-2 | 1 |  |  | - |  |  |
| 3-4 | 1·18 | 0·88-1·59 | 0·28 | - | - | - |
| 5+ | 1·21 | 0·91-1·62 | 0·20 | - | - | - |
| **Wealth index of households** |  |  |  |  |  |  |
| Poorest | 1 |  |  | 1 |  |  |
| Poorer | 1·44 | 0·85-2·51 | 0·18 | 1·35 | 0·76-2·43 | 0·31 |
| Middle | 2·27 | 1·43-3·73 | <0·01 | 1.99 | 1·21-3·40 | <0·01 |
| Richer | 3·58 | 2·35-5·68 | <0·001 | 2.75 | 1.73-4.59 | <0·001 |
| Richest | 8·07 | 5·38-12·67 | <0·001 | 5·03 | 3·14-8.43 | <0·001 |
| **Food security status of households** |  |  |  |  |  |  |
| Food insecure | 1 |  |  | 1 |  |  |
| Food secure | 1.96 | 1·68-2·30 | <0·001 | 1·16 | 0·97-1·39 | 0·10 |
| **Patterns of contraceptive use** |  |  |  |  |  |  |
| Never users | 1 |  |  | - |  |  |
| Currently users | 1·02 | 0·86-1·22 | 0·83 | - | - | - |
| Past users | 0·99 | 0·81-1·21 | 0·92 | - | - | - |
| **Menopausal status of women** |  |  |  |  |  |  |
| Not in menopause | 1 |  |  | - |  |  |
| In menopause | 0·92 | 0·81-1·05 | 0·21 | - | - | - |
| **Number of living children** |  |  |  |  |  |  |
| 0 | 1 |  |  | - |  |  |
| 1-2 | 1·48 | 1·18-1·86 | <0·01 | - | - | - |
| 3-4 | 1·59 | 1·26-2·02 | <0·001 | - | - | - |
| 5+ | 1·46 | 1·06-2·01 | <0·05 | - | - | - |
| **Current marital status** |  |  |  |  |  |  |
| Married and living with husband | 1 |  |  | - |  |  |
| Widowed/divorced/separated | 0·87 | 0·68-1·11 | 0·28 | - | - | - |
| **Educational status of women** |  |  |  |  |  |  |
| No education | 1 |  |  | 1 |  |  |
| Primary | 1·09 | 0·89-1·32 | 0·41 | 1·14 | 0·92-1·41 | 0·220 |
| Secondary | 1·61 | 1·36-1·92 | <0·001 | 1·39 | 1·13-1·71 | <0.01 |
| Higher | 2·32 | 1·90-2·85 | <0·001 | 1·43 | 1.11-1·83 | <0.01 |
| **Age (years) of women** |  |  |  |  |  |  |
| 18-22 | 1 |  |  | 1 |  |  |
| 23-27 | 1·99 | 1·59-2·52 | <0·001 | 1·94 | 1·53-2·46 | <0·001 |
| 28-32 | 2·74 | 2·18-3·46 | <0·001 | 2·61 | 2·05-3·32 | <0·001 |
| 33-37 | 3·30 | 2·60-4·19 | <0·001 | 3·14 | 2·45-4.04 | <0·001 |
| 38-42 | 3·73 | 2·95-4·74 | <0·001 | 3·95 | 3.07-5.10 | <0·001 |
| 43+ | 3·38 | 2·68-4·28 | <0·001 | 3.53 | 2·75-4·55 | <0·001 |
| **Age of women at first birth** |  |  |  |  |  |  |
| <=13 | 1 |  |  | - |  |  |
| 13-17 | 1·15 | 0·81-1·67 | 0·44 | - | - | - |
| 18-22 | 1·36 | 0·96-1·97 | 0·09 | - | - | - |
| >=23 | 2·05 | 1·40-3·02 | <0·001 | - | - | - |
| **Watching TV at least once a week** |  |  |  |  |  |  |
| No | 1 |  |  | 1 |  |  |
| Yes | 2·52 | 2.15-2·97 | <0·001 | 1·40 | 1·15-1·69 | <0·01 |
| **Occupation status of women** |  |  |  |  |  |  |
| Jobs that required manual labor | 1 |  |  | 1 |  |  |
| Jobs that required mostly sitting | 2·08 | 1·55-2·78 | <0·001 | 1·27 | 0·92-1·75 | 0·15 |
| Unemployed/Housewife | 1·74 | 1·44-2·10 | <0·001 | 1·39 | 1·14-1·71 | <0·01 |

**Table 12:** Odds ratio of logistic regression assessing the effects of selected variables on overweight and obesity status of rural women (excluded underweight women).

| **Variables with categories** | **Status of overweight and obesity of women** | | | | | |
| --- | --- | --- | --- | --- | --- | --- |
|  | **Simple logistic regression** | | | **Multiple logistic regression** | | |
| **Division** | **OR** | **95% CI** | **P value** | **OR** | **95% CI** | **P value** |
| Rangpur | 1 |  |  | 1 |  |  |
| Dhaka | 1·17 | 0·93-1·49 | 0·18 | 1·10 | 0·86-1·41 | 0·45 |
| Chittagong | 1·73 | 1·40-2·15 | <0·001 | 1·40 | 1·11-1·77 | <0·01 |
| Khulna | 1·72 | 1·39-2·14 | <0·001 | 1·39 | 1·11-1·75 | <0·01 |
| Rajshahi | 1·56 | 1·25-1·94 | <0·001 | 1·38 | 1·10-1·75 | <0·01 |
| Barisal | 1·11 | 0·86-1·42 | 0·42 | 1·04 | 0·79-1·36 | 0·78 |
| Sylhet | 1·32 | 1·03-1·68 | <0·05 | 1·05 | 0·80 -1·37 | 0·73 |
| **Number of household members** |  |  |  |  |  |  |
| 1-2 | 1 |  |  | 1 |  |  |
| 3-4 | 0·77 | 0·58-1·02 | 0·06 | 0·83 | 0·61-1·14 | 0·25 |
| 5+ | 0·71 | 0·55-0·94 | <0·01 | 0·73 | 0·54-1.00 | <0·05 |
| **Wealth index of households** |  |  |  |  |  |  |
| Poorest | 1 |  |  | 1 |  |  |
| Poorer | 1·16 | 0·92-1·47 | 0·22 | 1·08 | 0·84-1·39 | 0·56 |
| Middle | 1·96 | 1·58-2·44 | <0·001 | 1·57 | 1·23-2·01 | <0·001 |
| Richer | 3·44 | 2·79-4·26 | <0·001 | 2·42 | 1.87-3·15 | <0·001 |
| Richest | 5·45 | 4·37-6·84 | <0·001 | 3.45 | 2.58-4.64 | <0·001 |
| **Food security status of households** |  |  |  |  |  |  |
| Food insecure | 1 |  |  | 1 |  |  |
| Food secure | 1·77 | 1·55-2·03 | <0·001 | 1·19 | 1.03-1·39 | <0·05 |
| **Patterns of contraceptive use** |  |  |  |  |  |  |
| Never users | 1 |  |  | - |  |  |
| Currently users | 0·93 | 0·80-1·10 | 0·40 | - | - | - |
| Past users | 1·10 | 0·92-1·32 | 0·31 | - | - | - |
| **Menopausal status of women** |  |  |  |  |  |  |
| Not in menopause | 1 |  |  | - |  |  |
| In menopause | 0·85 | 0·75-0·96 | <0·05 | - | - | - |
| **Number of living children** |  |  |  |  |  |  |
| 0 | 1 |  |  | 1 |  |  |
| 1-2 | 1·00 | 0·79-1·26 | 0·98 | 0·75 | 0·57-0·98 | 0·03 |
| 3-4 | 1·02 | 0·80-1·29 | 0·90 | 0·69 | 0·52-0·94 | <0·05 |
| 5+ | 0·81 | 0·61-1·08 | 0·15 | 0·60 | 0·42-0·86 | <0·01 |
| **Current marital status** |  |  |  |  |  |  |
| Married and living with husband | 1 |  |  | - |  |  |
| Widowed/divorced/separated | 0·97 | 0·74-1·25 | 0·80 | - | - | - |
| **Educational status of women** |  |  |  |  |  |  |
| No education | 1 |  |  | 1 |  |  |
| Primary | 1·21 | 1·03-1·42 | <0·05 | 1.16 | 0·97-1·39 | 0·11 |
| Secondary | 1·74 | 1·49-2·03 | <0·001 | 1·42 | 1·17-1·74 | <0·01 |
| Higher | 2·67 | 2·09-3·41 | <0·001 | 1·46 | 1·09-1·96 | <0·05 |
| **Age (years) of women** |  |  |  |  |  |  |
| 18-22 | 1 |  |  | 1 |  |  |
| 23-27 | 1·91 | 1·53-2·40 | <0·001 | 2·21 | 1·74-2·82 | <0·001 |
| 28-32 | 2·59 | 2·08-3·25 | <0·001 | 3·22 | 2·50-4·15 | <0·001 |
| 33-37 | 2·39 | 1·90-3·02 | <0·001 | 3·20 | 2·44-4·21 | <0·001 |
| 38-42 | 2·47 | 1·96-3·13 | <0·001 | 3·71 | 2·78-4·96 | <0·001 |
| 43+ | 2·52 | 2·00-3·19 | <0·001 | 3·78 | 2·81-5.10 | <0·001 |
| **Age of women at first birth** |  |  |  |  |  |  |
| <=13 | 1 |  |  | - |  |  |
| 13-17 | 1·20 | 0·84-1·77 | 0·33 | - | - | - |
| 18-22 | 1·53 | 1·07-2·25 | <0·05 | - | - | - |
| >=23 | 1·88 | 1·26-2·89 | <0·01 | - | - | - |
| **Watching TV at least once a week** |  |  |  |  |  |  |
| No | 1 |  |  | 1 |  |  |
| Yes | 2·12 | 1·88-2·38 | <0·001 | 1·28 | 1.11-1·48 | <0.01 |
| **Occupation status of women** |  |  |  |  |  |  |
| Jobs that required manual labor | 1 |  |  | - |  |  |
| Jobs that required mostly sitting | 1·69 | 1·21-2·37 | <0·01 | - | - | - |
| Unemployed/Housewife | 1·15 | 0·92-1·45 | 0·23 | - | - | - |

**Table 13: Odds ratio of logistic regression assessing the impacts of selected variables on obesity (BMI≥30kg/m^2^) of rural women in Bangladesh**

|  | **Multiple Logistic Regression** | | |
| --- | --- | --- | --- |
| **Division** | **OR** | **95% CI** | **p value** |
| Rangpur | - |  |  |
| Dhaka | - | - | - |
| Chittagong | - | - | - |
| Khulna | - | - | - |
| Rajshahi | - | - | - |
| Barisal | - | - | - |
| Sylhet | - | - | - |
| **No· of household members** |  |  |  |
| 1-2 | 1 |  |  |
| 3-4 | 0.60 | 0.34-1.15 | 0.11 |
| 5+ | 0.42 | 0.23-0.79 | <0.01 |
| **Wealth index of households** |  |  |  |
| Poorest | 1 |  |  |
| Poorer | 1.81 | 0.58-2.49 | 0.65 |
| Middle | 1.80 | 0.93-3.65 | 0.09 |
| Richer | 3.49 | 1.84-7.04 | <0.001 |
| Richest | 8.18 | 4.24-16.78 | <0.001 |
| **Food-security status of households** |  |  |  |
| Food insecure | 1 |  |  |
| Food secure | 1.61 | 1.08-2.48 | <0.05 |
| **Patterns of contraceptive use** |  |  |  |
| Never users | - |  |  |
| Currently users | - | - | - |
| Past users | - | - | - |
| **Menopausal status of women** |  |  |  |
| Not in menopause | 1 |  |  |
| In menopause | 1.30 | 0.93-1.80 | 0.12 |
| **Number of living children** |  |  |  |
| 0 | 1 |  |  |
| 1-2 | 0.49 | 0.28-0.90 | <0.05 |
| 3-4 | 0.50 | 0.27-0.97 | <0.05 |
| 5+ | 0.65 | 0.31-1.40 | 0.26 |
| **Current marital status** |  |  |  |
| Married and living with husband | - | - | - |
| Widowed/divorced/separated | - | - | - |
| **Educational status of women** |  |  |  |
| No education | - | - | - |
| Primary | - | - | - |
| Secondary | - | - | - |
| Higher | - | - | - |
| **Age (years) of women** |  |  |  |
| 18-22 | 1 |  |  |
| 23-27 | 4.83 | 2.28-11.48 | <0.001 |
| 28-32 | 6.16 | 2.87-14.85 | <0.001 |
| 33-37 | 9.50 | 4.41-22.96 | <0.001 |
| 38-42 | 7.45 | 3.33-18.45 | <0.001 |
| 43+ | 6.71 | 2.96-16.82 | <0.001 |
| **Age (years) of women at first birth** |  |  |  |
| <=13 | - |  |  |
| 13-17 | - | - | - |
| 18-22 | - | - | - |
| >=23 | - | - | - |
| **Watching TV at least once a week** |  |  |  |
| No | 1 | - | - |
| Yes | 1.48 | 1.05-2.11 | <0.05 |
| **Occupation status of women** |  |  |  |
| Jobs that required manual labor | - |  |  |
| Jobs that required mostly sitting | - | - | - |
| Unemployed/housewives | - | - | - |

**Table 14: Odds ratio of logistic regression assessing the impacts of selected variables on obesity (BMI≥30kg/m^2^) of urban women in Bangladesh**

|  | **Multiple Logistic Regression** | | |
| --- | --- | --- | --- |
| **Division** | **OR** | **95% CI** | **p value** |
| Rangpur | - | - | - |
| Dhaka | - | - | - |
| Chittagong | - | - | - |
| Khulna | - | - | - |
| Rajshahi | - | - | - |
| Barisal | - | - | - |
| Sylhet | - | - | - |
| **No· of household members** |  |  |  |
| 1-2 | - | - | - |
| 3-4 | - | - | - |
| 5+ | - | - | - |
| **Wealth index of households** |  |  |  |
| Poorest | 1 |  |  |
| Poorer | 1.68 | 0.16-36.35 | 0.67 |
| Middle | 5.10 | 0.96-93.97 | 0.12 |
| Richer | 9.96 | 2.13-177.54 | <0.05 |
| Richest | 20.13 | 4.346 358.241 | <0.01 |
| **Food-security status of households** |  |  |  |
| Food insecure | - | - | - |
| Food secure | - | - | - |
| **Patterns of contraceptive use** |  |  |  |
| Never users | - |  |  |
| Currently users | - | - | - |
| Past users | - | - | - |
| **Menopausal status of women** |  |  |  |
| Not in menopause | 1 |  |  |
| In menopause | 1.22 | 0.94-1.57 | 0.13 |
| **Number of living children** |  |  |  |
| 0 | - | - | - |
| 1-2 | - | - | - |
| 3-4 | - | - | - |
| 5+ | - | - | - |
| **Current marital status** | - |  |  |
| Married and living with husband | - | - | - |
| Widowed/divorced/separated | - | - | - |
| **Educational status of women** |  |  |  |
| No education | 1 |  |  |
| Primary | 1.02 | 0.68-1.53 | 0.93 |
| Secondary | 1.44 | 1.01-2.10 | 0.05 |
| Higher | 1.35 | 0.89-2.07 | 0.16 |
| **Age (years) of women** |  |  |  |
| 18-22 | 1 |  |  |
| 23-27 | 1.41 | 0.86-2.36 | 0.19 |
| 28-32 | 2.56 | 1.61-4.17 | <0.001 |
| 33-37 | 3.87 | 2.44-6.33 | <0.001 |
| 38-42 | 3.77 | 2.36-6.19 | <0.001 |
| 43+ | 3.74 | 2.35-6.13 | <0.001 |
| **Age (years) of women at first birth** |  |  |  |
| <=13 | - |  |  |
| 13-17 | - | - | - |
| 18-22 | - | - | - |
| >=23 | - | - | - |
| **Watching TV at least once a week** |  |  |  |
| No | 1 |  |  |
| Yes | 2.05 | 1.36-3.19 | <0.001 |
| **Occupation status of women** |  |  |  |
| Jobs that required manual labor | 1 |  |  |
| Jobs that required mostly sitting | 1.60 | 0.87-2.94 | 0.13 |
| Unemployed/housewives | 1.87 | 1.24-2.95 | <0.01 |

**Table 15: Generalized variance inflation (GVIF) values of final models of Rural and Urban women**

|  | **Multiple Logistic Regression (Rural)** | **Multiple Logistic Regression (Urban)** |
| --- | --- | --- |
|  | **GVIF** | **GVIF** |
| **Division** | 1.21 | 1.14 |
| **No· of household members** | 1.34 | - |
| **Wealth index of households** | 1.87 | 1.59 |
| **Food-security status of households** | 1.18 | 1.17 |
| **Number of living children** | 2.15 | - |
| **Current marital status** | 1.07 | - |
| **Educational status of women** | 1.77 | 1.56 |
| **Age (years) of women** | 2.06 | 1.17 |
| **Watching TV at least once a week** | 1.35 | 1.25 |
| **Occupation status of women** | - | 1.14 |
